# Influence of BMI percentile on craniofacial morphology and development in adolescents,Part II: elevated BMI is associated with larger final facial dimensions

**DOI:** 10.1093/ejo/cjad043

**Published:** 2023-11-02

**Authors:** Steven Hancock, Andrea Carmack, Mallory Kocher, Erika Rezende Silva, Taylor Sulkowski, Eleanor Nanney, Christina Graves, Kelly Mitchell, Laura Anne Jacox

**Affiliations:** Division of Craniofacial and Surgical Care, Orthodontics Group, Adams School of Dentistry, University of North Carolina, 270 Brauer Hall, CB#270, Chapel Hill, NC 25799-7450, United States; Department of Biostatistics, Gillings School of Global Public Health, University of North Carolina at Chapel Hill, 135 Dauer Drive, 3101 McGavran, Chapel Hill, NC 27599, United States; DDS Program, Adams School of Dentistry, University of North Carolina, 270 Brauer Hall, CB#270, Chapel Hill, NC 25799-7450, United States; Oral and Craniofacial Biomedicine Program, Adams School of Dentistry, University of North Carolina, 365 S Columbia St, Chapel Hill, NC 25799-7450, United States; Division of Oral and Craniofacial Health Sciences, Adams School of Dentistry, University of North Carolina, CB #7455, Chapel Hill, NC 27599-7450, United States; Division of Craniofacial and Surgical Care, Orthodontics Group, Adams School of Dentistry, University of North Carolina, 270 Brauer Hall, CB#270, Chapel Hill, NC 25799-7450, United States; Division of Craniofacial and Surgical Care, Orthodontics Group, Adams School of Dentistry, University of North Carolina, 270 Brauer Hall, CB#270, Chapel Hill, NC 25799-7450, United States; Division of Oral and Craniofacial Health Sciences, Adams School of Dentistry, University of North Carolina, CB #7455, Chapel Hill, NC 27599-7450, United States; Division of Craniofacial and Surgical Care, Orthodontics Group, Adams School of Dentistry, University of North Carolina, 270 Brauer Hall, CB#270, Chapel Hill, NC 25799-7450, United States; Division of Craniofacial and Surgical Care, Orthodontics Group, Adams School of Dentistry, University of North Carolina, 270 Brauer Hall, CB#270, Chapel Hill, NC 25799-7450, United States; Division of Oral and Craniofacial Health Sciences, Adams School of Dentistry, University of North Carolina, CB #7455, Chapel Hill, NC 27599-7450, United States

**Keywords:** body mass index, obesity, craniofacial morphology, orthodontics, craniofacial development, development, cervical vertebrae maturation, bimaxillary prognathism, treatment timing, dentofacial disharmony, growth, malocclusion

## Abstract

**Background:**

Prevalence of adolescent obesity has markedly increased from 5.2% in 1974 to 19.7% in 2021. Understanding the impacts of obesity is important to orthodontists, as growth acceleration and greater pre-pubertal facial dimensions are seen in children with elevated body mass index (BMI).

**Methods:**

To identify whether adolescent obesity shifts the timing and rate of craniofacial growth resulting in larger post-treatment dimensions, we evaluated cephalometric outcomes in overweight/obese (BMI > 85%, *n* = 168) and normal weight (*n* = 158) adolescents (*N* = 326 total). Cephalometric measurements were obtained from pre- and post-treatment records to measure growth rates and final dimensions and were statistically evaluated with repeated measures analysis of variance and linear regression models.

**Results:**

Overweight and obese adolescents began and finished treatment with significantly larger, bimaxillary prognathic craniofacial dimensions, with elevated mandibular length [articulare-gnathion (Ar-Gn)], maxillary length [condylion-anterior nasal spine (Co-ANS), posterior nasal spine-ANS (PNS-ANS)], and anterior lower face height (ANS-Me), suggesting overweight children grow more overall. However, there was no difference between weight cohorts in the amount of cephalometric change during treatment, and regression analyses demonstrated no correlation between change in growth during treatment and BMI. BMI percentile was a significant linear predictor (*P* < 0.05) for cephalometric post-treatment outcomes, including Ar-Gn, Co-ANS, ANS-Me, upper face height percentage (UFH:total FH, inverse relationship), lower face height percentage (LFH:total FH), sella-nasion-A-point (SNA), and SN-B-point (SNB).

**Limitations:**

The study is retrospective.

**Conclusions:**

Growth begins earlier in overweight and obese adolescents and continues at a rate similar to normal-weight children during orthodontic treatment, resulting in larger final skeletal dimensions. Orthodontics could begin earlier in overweight patients to time care with growth, and clinicians can anticipate that overweight/obese patients will finish treatment with proportionally larger, bimaxillary-prognathic craniofacial dimensions.

## Introduction

The obesity epidemic continues to grow with an ever-rising prevalence of adolescent and childhood obesity [1]. According to the National Health and Nutrition Examination Survey (NHANES) published in 2021, a striking 19.7% of children and adolescents aged 2–19 years are obese, compared to 1974 when 10.2% of children were overweight and 5.2% were obese [2]. Children who are overweight and obese often experience negative psychological sequelae, with increased anxiety, social isolation, and lower health-related quality of life [3]. Lower self-esteem and social isolation may influence overweight children’s attitudes towards orthodontic treatment [4]. These children may disregard their orthodontic problems and not seek treatment or may become hyper-aware of their malocclusion and develop unrealistic expectations for orthodontic care [4]. Obesity also has profound effects on the physical health and growth status of children and adults. Significant sequelae include increased risk of type 2 diabetes, cardiovascular disease, joint issues, asthma, reduced growth hormone secretion, and sleep apnea, among others [5–7]. Many studies link obesity to altered skeletal and dental development [8–12]. Mack *et al.* found a significant increase in the cervical vertebral maturation (CVM) stage in patients with an increased body mass index (BMI), indicating greater skeletal maturity, consistent with our findings in Danze *et al.* [9, 10]. Increased leptin and sex hormone levels may produce accelerated epiphyseal maturation and skeletal pubertal growth seen in obese children [13]. Dental development is also accelerated, with overweight and obese children demonstrating a 1- to 2-year dental age increase relative to chronological age, with a 0.005-year dental age increase correlating with each BMI percentile [9, 10].

Along with skeletal and dental maturity, the interaction of BMI and craniofacial morphology has been examined at the start of orthodontic treatment. Olszewska *et al.* found patients with higher BMI exhibited greater mandibular length (Cd-Gn), corpus length (Go-Pg), midfacial length (Cd-A), and smaller anterior face height and anterior-posterior angular measurements when compared to normal-weight subjects [11]. Obesity was associated with increased bimaxillary prognathism and greater craniofacial dimensions in Sadeghianrizi *et al.* [12]. Similarly, in our earlier study, we found that obesity was associated with bimaxillary prognathism and greater craniofacial dimensions including mandibular length (Ar-Gn), maxillary length (Co–ANS), posterior (S-Go) and anterior face heights (N-Me) at treatment start [10].

Prior studies evaluated a single time point using pre-treatment records, without determining the impacts of elevated BMI at the end of orthodontic treatment. Therefore, it is unclear if larger facial dimensions at the start of treatment are reflective of an earlier start and finish of craniofacial growth, where the final facial dimensions of overweight/obese children are similar to kids with normal weights. Alternatively, greater facial dimensions at the start of treatment could be associated with both earlier growth and longer growth overall, such that the final skeletal dimensions of overweight/obese patients end up larger than normal-weight adolescents at treatment end. Furthermore, it is unknown how the rate of growth during treatment varies between normal weight and overweight/obese adolescents. To address these points, we extended our cross-sectional study of a large, diverse adolescent patient pool, described in Danze *et al.* to include post-treatment records with the goal of determining the rate of growth during treatment and the final craniofacial dimensions of adolescent patients with normal and elevated BMIs [10]. Understanding the long-term sequelae of elevated BMI on craniofacial growth holds great relevance to orthodontic treatment planning and timing.

## Methods

### Sample preparation

Retrospective data were obtained from a patient database at the University of North Carolina (UNC) Adams School of Dentistry Orthodontic Clinic from September 2014 to September 2018. An initial sample of 400 consecutively treated individuals (200 normal weight patients, 200 obese/overweight patients) was derived from the Danze *et al.* study after 1381 potential subjects’ records were screened and 981 were excluded for underweight BMI, out-of-range age, growth-impacting medical conditions, congenital anomalies visible on the radiograph, or low visibility of cervical vertebra on lateral cephalograms [10]. Individuals were evaluated against updated enrollment criteria and selected for inclusion (Table 1). Of the 400 individuals included in the Danze *et al.* study, 74 were excluded due to: continued treatment (n=10), patients not reaching growth cessation at CVM 5–6 (n=9), incomplete records (n=52), or patients who underwent surgical correction (n=3). Pre-treatment BMI was defined as recommended by the United States Centers for Disease Control and Prevention (CDC) (BMI = weight (kg)/height (m)^2^), overweight: BMI 25–29.9; obese: BMI ≥ 30). For children, growth charts are used to define weight status; children between the 85th and 95th percentile are considered overweight while children at the 95th percentile or greater are considered obese [14]. Ethics approval was granted by the Institutional Review Board of the University of North Carolina at Chapel Hill (IRB #21-782).

**Table 1.** Enrollment criteria.

Inclusion criteria	Exclusion criteria
- Age greater than or equal to 8 years but less than 15 years at the time of pre-treatment records (T1) - Pre-treatment and post-treatment lateral cephalometric radiographs of adequate diagnostic quality - Pre-treatment BMI percentile between 5 and 100 (normal weight, overweight, and obese) - Post-treatment cervical vertebrae maturation stage of CVM 5 or 6 - Patients must have started and completed treatment at UNC Adams School of Dentistry Orthodontics Department with full initial and final records including cephalometric radiographs	- Second, third, and fourth cervical vertebrae not clearly visible on the lateral cephalometric radiograph - Post-treatment CVM stage of 4 or less - Presence of congenital anomalies of the second, third, or fourth vertebrae - Congenital craniofacial anomalies visible in the Cephalometric radiographs - Significant medical conditions that would affect physical growth and development - Craniofacial defects - BMI percentile less than 5 (underweight) - Patients who underwent surgery prior to post-pubertal lateral cephalometric radiograph

### Cephalometric analysis

Post-treatment radiographs were taken and cephalometric radiographs were traced by a single examiner, as previously described [10]. Cephalometric dimensions include:

- linear measures: mandibular length [articulare-gnathion (Ar-Gn), gonion-pogonion (Go-Pg)], ­maxillary length [condylion-anterior nasal spine (Co-ANS), posterior nasal spine-anterior nasal spine (PNS-ANS)], posterior face height [sella-gonion (S-Go)], anterior lower face height [ANS-menton (ANS-Me)], nasion-menton (N-Me), nasion-ANS (N-ANS), and sella-nasion (S-N);
- facial proportions: upper face height percentage [upper face height: total face height (UFH:total FH)], lower face height percentage [lower face height: total face height (LFH:total FH)]; and
- angular measures: sella-nasion-A-point (SNA), sella-nasion-B-point (SNB), A-point-nasion-B point (ANB), mandibular plane angle [sella-nasion-gonion-gnathion (SN-GoGn)], and pogonial projection [sella-nasion-pogonion (SN-Pg)].

A clinically significant difference in mean linear measurement was defined *a priori* as a difference of 2 mm or greater between groups [10]. For angular measurements, clinical significance was defined as a difference of 2° or more between mean values [15]. The threshold of significance for all statistical analyses is a *P* value less than 0.05.

To evaluate intraclass correlation agreement, 20 subjects from each group (*n* = 40 total) were selected by a random number generator; the same examiner re-traced these subjects’ post-treatment radiographs for a concordance correlation reliability test. A concordance correlation reliability test was used to evaluate intra-rater cephalometric tracing reliability (Supplementary Table S1).

### Statistical analyses

The sample consisted of all 326 individuals and 16 outcomes were measured on each individual at two time points (with no missing data). Of the 326 participants, 158 had a normal BMI and 168 were classified as being overweight or obese. The primary exposure of interest was BMI, dichotomized as normal versus overweight/obese, and also treated as a continuous variable using BMI percentile. Outcomes were assessed both post-treatment and as the change in each outcome between time points. We reported descriptive statistics on demographic variables stratified by BMI groups. Chi-squared tests determined whether BMI groups differed in categorical variables. For all tests, raw *P* values are reported alongside Bonferroni-adjusted *P* values (for multiple testing).

General linear models adjusted for gender, age, race, and ethnicity were used to determine statistically different growth quantities between the two study groups (normal and obese/overweight) from pre- to post-treatment records. We also utilized repeated measures analysis of variance (ANOVA) adjusted for gender, age, race, and ethnicity to evaluate differences in craniofacial dimensions by BMI group at treatment end (time point 2) and the rate of change over time between groups. The main effects of time and BMI were included in the repeated measure ANOVA model, as well as the interaction between time and BMI.

We performed linear regression adjusting for gender, race, and ethnicity to determine the significance of BMI percentile as a predictor of each craniofacial dimension post-treatment and of the change in each craniofacial dimension. Age was not adjusted for, consistent with prior studies [10].

## Results

### Sample description

The sample consists of 326 adolescents who underwent comprehensive orthodontic treatment, with complete pre-treatment (T1, initial) and post-treatment (T2, final) records. Adolescents ranged between 8 and 14 years of age pre-treatment (T1) and 12–18 years of age post-treatment (T2) (Table 2). The mean age at T1 was 12.4 years for the normal BMI group and 12.3 years for the overweight/obese group (*P* = 0.274), while the mean age at T2 was 14.8 years for the normal BMI group and 14.7 years for the overweight/obese group (*P* = 0.910). Of the patients, 158 had a normal BMI and 168 were classified as being overweight or obese. The distributions of age and gender were not significantly different between BMI groups (Table 2). At T1, overweight/obese subjects had a significantly advanced CVM staging relative to the normal BMI subjects, as previously reported (Table 2) [10, 16]. However, there was no statistically significant difference in CVM stage distribution at T2 on treatment conclusion (Table 2). At T2, 82.9% of normal weight subjects were at CVM5 and 17.1% were at CVM6, while 78.6% of overweight/obese subjects were at CVM5 and 21.4% were at CVM6 (*P* = 0.321). Similarly, there was no significant difference in treatment length nor distribution of angle classification between BMI cohorts at T1 and T2 (Table 2). A larger portion of the overweight/obese population was African American compared to the normal BMI group; however, statistical adjustments were made to account for age, gender, race, and ethnicity. The concordance coefficient reliability values for cephalometric tracing measurements were all above 0.90, indicating high reliability (Supplementary Table S1).

**Table 2.** Sample demographics.

		Normal weight 5% < BMI < 85% (*n* = 158)		Overweight to obese BMI > 85% (*n* = 168)		*P* value
		Frequency	Percentage	Frequency	Percentage	
Race and ethnicity						0.009*^
	African American	10	3.1%	24	7.5%	
	Caucasian	104	31.9%	88	27.0%	
	Hispanic	32	9.8%	49	15.0%	
	Other***	12	3.7%	7	2.2%	
Gender						0.548*
	Female	86	26.4%	97	29.8%	
	Male	72	22.1%	71	21.8%	
Age at T1						0.274*
	8yo	1	0.3%	2	0.6%	
	9yo	4	1.2%	1	0.3%	
	10yo	13	4.0%	7	2.2%	
	11yo	17	5.2%	29	8.9%	
	12yo	41	12.6%	49	15.0%	
	13yo	48	14.7%	48	14.7%	
	14yo	34	10.4%	32	9.8%	
	Mean	12.4	NA	12.3	NA	
	SD	1.3	NA	1.2	NA	
Age at T2						0.910*
	12yo	3	0.9%	4	1.2%	
	13yo	17	5.3%	25	7.7%	
	14yo	39	12.0%	44	13.6%	
	15yo	55	16.9%	52	16.0%	
	16yo	34	10.5%	31	9.6%	
	17yo	8	2.5%	10	3.1%	
	18yo	2	0.6%	2	0.6%	
	Mean	14.8	NA	14.7	NA	
	SD	1.2	NA	1.2	NA	
Phase II treatment length						0.901**
	N	134	84.8%	154	91.7%	
	Min	10 mo.	NA	10 mo.	NA	
	Max	49 mo.	NA	48 mo.	NA	
	Mean	23.6 mo.	NA	23.7 mo.	NA	
	SD	6.8 mo.	NA	7.2 mo.	NA	
T1 angle^^ classification						0.094*
	Class I	74	46.8%	56	33.4%	
	Mild Class II	57	36.1%	76	45.2%	
	Severe Class II	18	11.4%	22	13.1%	
	Class III	9	5.7%	14	8.3%	
T2 angle^^ classification						0.145*
	Class I	80	50.6%	68	40.5%	
	Mild Class II	45	28.5%	68	40.5%	
	Severe Class II	14	8.9%	13	7.7%	
	Class III	19	12.0%	19	11.3%	
CVM at T1						<.001*^
	CVM1	55	34.8%	19	11.3%	
	CVM2	40	25.3%	40	23.8%	
	CVM3	38	24.1%	45	26.8%	
	CVM4	25	15.8%	54	32.1%	
	CVM5	0	0	10	6.0%	
CVM at T2						0.321*
	CVM2	0	0	0	0	
	CVM3	0	0	0	0	
	CVM4	5	Excluded	4	Excluded	
	CVM5	131	82.9%	132	78.6%	
	CVM6	27	17.1%	36	21.4%	

*P values calculated by chi-squared test. Chi-square tests were performed to compare the frequencies of race and gender in each BMI group.

**P value calculated by independent T-test.

***The Other category can include Asian American, Native American, Alaska Native, Native Hawaiian, and other Pacific Islanders. Our other sample only included subjects who were Asian American and Native American and were grouped because of their small number.

^*P* value < 0.05 threshold for significance. Indicates that race/ethnicity and BMI percentile are not independent.

^^Angle classification was defined by ANB angle to reflect skeletal relationships. ANB > 6°: Severe Class II. ANB ranging from 3.01° to 6°: Mild Class II. ANB ranging from 0.01° to 3°: Class I. ANB of ≥ 0°: Class III.

### Craniofacial growth during treatment

The interaction between BMI and time was not significant for changes in craniofacial dimensions during treatment, indicating that growth rates were similar between normal weight and elevated BMI cohorts. The total change in cephalometric dimensions between pre- and post-treatment records (T2-T1), when comparing groups, differed only for the S-Go (mm) (*P* = 0.029) without multiple testing adjustment, and showed no difference after Bonferroni adjustment, indicating the change in growth was similar during treatment for normal and overweight/obese adolescents (Supplementary Table S2). Among both BMI cohorts, 9 of the 16 cephalometric outcomes significantly increased between treatment start and end indicative of growth, including mandibular length (Ar-Gn, Go-Pg), maxillary length (Co-ANS, PNS-ANS), posterior face height (S-Go), and lower face height (ANS-Me), S-N, N-Me, and N-ANS, while UFH percentage (UFH:total FH), ANB (deg), and SNA (deg) decreased between time points 1 and 2 (Supplementary Table S3). Pre-treatment measures including N-Me (mm), SNA (deg), SNB (deg), and SN-Pg (deg) were larger in the overweight/obese group compared to the normal group but were not significantly different in post-treatment records (Table 3).

**Table 3.** Mean (SD) of craniofacial dimensions at end of treatment (T2) by weight group.

	Normal weight BMI group (*n* = 148)				Overweight/Obese BMI group (*n* = 168)						
Variable^	Mean	SD	Min	Max	Mean	SD	Min	Max	*P* value	Bonf. *P* value	Mean diff.***
Ar-Gn (mm)	117.0	7.7	99.7	137.4	120.6	8.1	103.8	147.5	<.001*	<.002**	−3.52
Go-Pg (mm)	77.3	5.7	63.1	92.1	78.5	5.7	66.9	94.8	0.107	1.000	−0.91
Co-ANS (mm)	90.9	5.5	74.8	109.9	92.8	4.7	81.7	105.6	<.001*	<.002**	−1.91
PNS-ANS (mm)	53.4	3.5	45.4	61.6	54.5	3.0	45.5	62.7	<.001*	<.002**	−1.16
S-N (mm)	72.8	4.2	61.7	85.9	72.9	4.2	63.4	83.2	0.161	1.000	−0.52
S-Go (mm)	81.8	6.4	62.0	95.4	82.4	7.3	67.1	99.1	0.060	0.960	−1.14
N-Me (mm)	122.4	8.2	106.0	145.5	124.3	8.3	102.2	147.4	0.008*	0.128	−2.01
N-ANS (mm)	55.6	3.5	44.8	65.2	55.6	3.7	44.5	65.7	0.803	1.000	−0.09
ANS-Me (mm)	68.3	6.5	54.8	85.6	70.4	6.1	52.1	87.4	0.003*	0.048**	−1.89
UFH:total FH (%)	0.45	0.02	0.40	0.52	0.45	0.02	0.39	0.52	0.008	0.128	0.0069
LFH:total FH (%)	0.56	0.03	0.49	0.61	0.57	0.02	0.51	0.64	0.017	0.272	−0.006
SNA (deg)	81.2	3.6	70.4	92.1	82.4	3.7	74.8	95.0	0.045*	0.720	−0.79
SNB (deg)	78.6	3.8	69.0	90.6	79.6	3.6	71.1	92.3	0.033*	0.528	−0.88
ANB (deg)	2.7	2.4	−2.7	11.4	2.8	2.5	−5.5	8.9	0.731	1.000	0.09
SN-Pg (deg)	79.6	4.1	69.5	92.5	80.2	3.6	71.1	90.8	0.113	1.000	−0.68
SN-GoGn (deg)	30.5	6.1	13.2	51.7	31.3	5.4	16.2	46.6	0.600	1.000	−0.33

^Variable abbreviations: Ar, articulare; Gn, gnathion; Go, gonion; Pg, pogonion; Co, condylion; ANS, anterior nasal spine; PNS, posterior nasal spine; S, sella; N, nasion; Me, menton; UFH, upper face height; LFH, lower face height; SNA, sella-nasion-A point; SNB, sella-nasion-B point; ANB, A point-nasion-B point.

*Indicates significance (*P* value < 0.05) by ANOVA prior to multiple testing adjustment. Please interpret at your discretion.

**Indicates significance (*P* value < 0.05) by ANOVA after Bonferroni adjustment.

***A negative mean difference indicates that the overweight/obese individuals had a larger mean value than the normal weight individuals.

**Figure 1.**
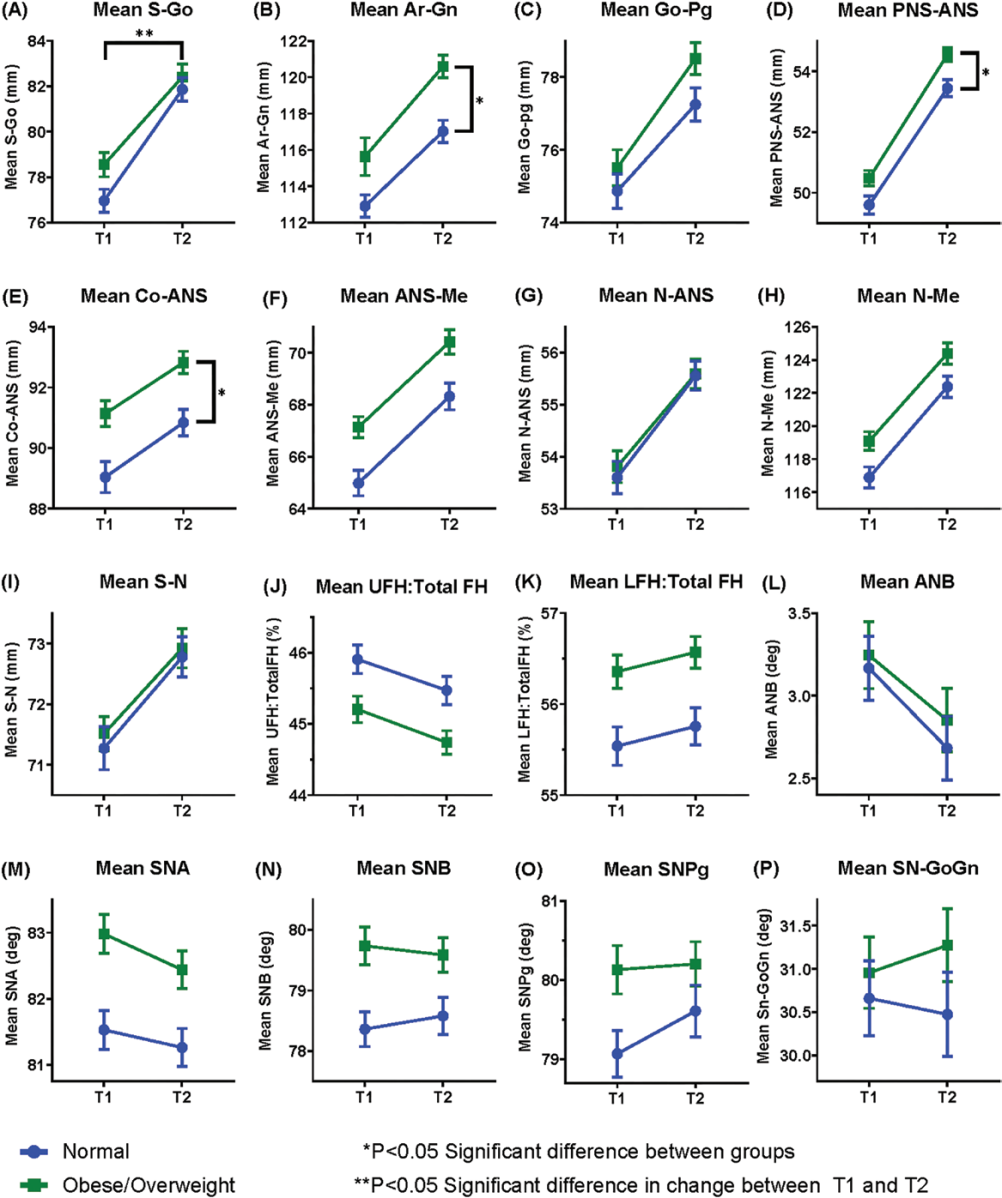
Pre- and post-treatment craniofacial dimensions of normal weight and overweight/obese children. Mean values and standard errors (S.E.) of linear (mm) and angular (degree) cephalometric measurements, including (a) S-Go, (b) Ar-Gn, (c) Go-Pg, (d) PNS-ANS, (e) Co-ANS, (f) ANS-Me, anterior lower face height, (g) N-ANS, (h) N-Me, (i) S-N, (j) UFH:total FH, (k) LFH:total FH, (l) ANB, (m) SNA, (n) SNB, (o) SN-Pg, and (p) SN-GoGn. Time point 1 (T1): pre-treatment records. Time point 2 (T2): post-treatment records. Normal weight mean values are blue circles and overweight/obese mean values are green squares. *P < 0.05 significance threshold for differences between groups in a craniofacial dimension at T2, with Bonferroni adjustment. **P < 0.05 significance threshold for differences between groups in the change in a craniofacial dimension between T2 and T1, without Bonferroni adjustment. Data are given in Table 3 and Supplementary Table S2.

Notably, at the conclusion of treatment, mandibular length (Ar-Gn), maxillary length (Co-ANS, PNS-ANS), and lower face height (ANS-Me) were significantly larger in the overweight/obese cohort than the normal weight cohort, suggesting overweight children grow more in these dimensions (Table 3, Fig. 1). There was a clinically significant increase of greater than 2 mm in lower face height (ANS-Me) and mandibular length (Ar-Gn) among the overweight/obese cohort relative to normal BMI participants. Data indicate that adolescents of both BMI cohorts grow during orthodontic treatment at similar rates, though the elevated BMI group began and finished treatment with larger craniofacial dimensions.

### Linear correlations between BMI and growth

Linear regression analysis showed no correlation between change in growth during treatment and BMI percentile, after Bonferroni adjustment (Supplementary Table S4). Before Bonferroni adjustments, the change in posterior face height (S-Go) and N-ANS had a positive linear correlation with increasing BMI (Supplementary Table S4). Findings indicate no strong linear correlation between the rate of growth and BMI percentile during treatment.

**Figure 2.**
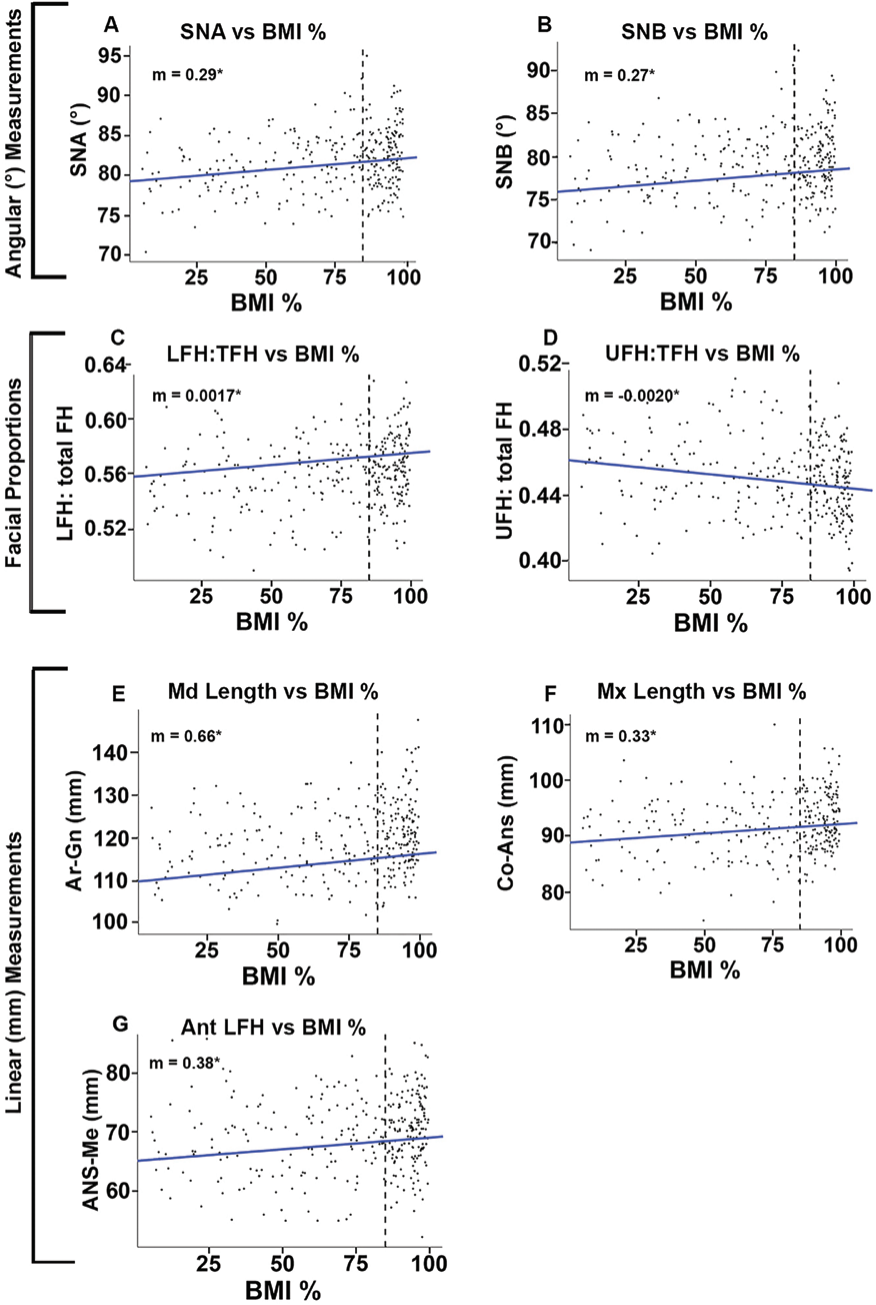
Significant linear regression plots of post-treatment craniofacial dimensions as a function of body mass index (BMI) percentile. (a) Sella-nasion-A point (SNA) versus BMI%; (b) sella-nasion-B point (SNB) versus BMI%; (c) lower face height percentage (LFH:total FH) versus BMI%; (d) upper face height percentage (UFH:total FH) versus BMI%; mandibular length (Ar-Gn) versus BMI%; maxillary length (Co-ANS) versus BMI%; anterior lower face height (ANS-Me) versus BMI%. Dotted line: 85% BMI percentile that is the dividing line between normal weight (BMI% < 85%) and overweight/obese (BMI% ≥ 85%). Data adjusted for age, gender, race, and ethnicity. All linear relationships shown are statistically significant (*P* < 0.05), with Bonferroni adjustment. Data are given in Table 4.

BMI percentile was a significant linear predictor for seven cephalometric post-treatment outcomes, after Bonferroni adjustment, including mandibular length (Ar-Gn (mm)), maxillary length (Co-ANS (mm)), anterior lower face height (ANS-Me (mm)), UFH:total FH, LFH:total FH, SNA (deg), and SNA (deg) (Table 4, Fig. 2). Before multiple testing adjustments, Go-Pg (mm), PNS-ANS (mm), S-Go (mm), N-Me (mm), and SN-Pg (deg) also demonstrated significant linear trends as a function of BMI percentile. All these post-treatment cephalometric measures had a positive linear relationship with BMI, with the post-treatment dimensions increasing linearly with rising BMI percentile, except for UFH percentage (UFH:total FH), which had an inverse relationship; UFH percentage decreased linearly with increasing BMI, as LFH percentage increased (Table 4, Fig. 2). Mandibular unit length (Ar-Gn) had a change of 0.66 mm, maxillary unit length (Co-ANS) had a change of 0.33 mm, and lower face height (ANS-Me) had a change of 0.38 mm, for every 10% change in BMI percentile (Table 4); these three measures had clinically significant differences between groups at time point 2 (Table 3).

**Table 4.** Significant linear regression slopes of end of treatment (T2) craniofacial dimensions as a function of BMI percentile.

Variable	Slope^	*P* value	Bonf. *P* value
Ar-Gn (mm)	0.66	<.001*	<.002**
Go-Pg (mm)	0.25	0.019	0.304
Co-ANS (mm)	0.33	<.001*	<.002**
PNS-ANS (mm)	0.19	0.004	0.064
S-N (mm)	−0.00	0.994	1.000
S-Go (mm)	0.29	0.014	0.224
N-Me (mm)	0.32	0.027	0.432
N-ANS (mm)	−0.06	0.359	1.000
ANS-Me (mm)	0.38	0.002*	0.032**
UFH:total FH	−0.0020	<.001*	<.002**
LFH:total FH	0.0017	<.001*	<.002**
SNA (deg)	0.29	<.001*	<.002**
SNB (deg)	0.27	<.001*	<.002**
ANB (deg)	0.02	0.660	1.000
SN-Pg	0.24	0.004*	0.064
SN-GoGn (deg)	−0.05	0.684	1.000

*Indicates significant linear relationship between craniofacial dimension and BMI% (*P* value < 0.05) prior to multiple testing adjustment. Please interpret at your discretion.

**Indicates significant linear relationship between craniofacial dimension and BMI% (*P* value < 0.05) after Bonferroni adjustment.

^Slopes reflect the average change in the craniofacial outcome per 10% increase in BMI percentile.

## Discussion

Our data are the first to indicate that in overweight/obese children, craniofacial growth begins earlier, continues throughout treatment at a rate similar to normal-weight adolescents, and results in larger overall skeletal dimensions at treatment conclusion. A clinically and statistically significant increase was observed in post-treatment maxillary and mandibular length among the overweight/obese patient cohort, indicative of a proportional, bimaxillary prognathic presentation. Linear relationships were found between increasing BMI and several post-treatment cephalometric outcomes. At treatment start (T1), elevated BMI patients were significantly advanced by CVM stage relative to normal weight subjects at the same mean age, while at treatment conclusion (T2), there was no difference in CVM stage or mean age. These results suggest that adolescents with elevated BMI start growing earlier, continue growing throughout treatment at a similar rate, and therefore grow more in total than patients with normal BMI. Altogether these data provide valuable insights into the longitudinal effects of elevated BMI on craniofacial growth during and after orthodontic treatment.

Our pre- and post-treatment findings are consistent with prior studies which found that elevated BMI is associated with larger craniofacial dimensions and increased prevalence of bimaxillary prognathism at the start of treatment [9–11, 17]. Specifically, Olszewska *et al.* found a greater pre-treatment mandibular length, maxillary length, and anterior face height in overweight children [11]. Ohrn *et al.* also identified a larger mean mandibular length, jaw prognathism, and smaller upper anterior face height among overweight/obese adolescents [17]. Similarly, our initial and final cephalometric measures show a significantly larger mean mandibular length, maxillary length, anterior face height, SNA, and SNB in children with elevated BMI. Danze *et al.* and Olszeska *et al.* found a difference in posterior face height or SN-Pg between weight cohorts at the treatment start [10, 11]. However, in the Danze *et al.* patients’ final records, shown here, there was no significant difference in posterior face height or SN-Pg, suggesting that normal-weight individuals experience later growth in the posterior vertical dimension of the mandible and pogonial projection, when compared to the overweight/obese group (Fig. 1, Table 3) [10]. Post-treatment measures clarify how growth is expressed during orthodontic treatment in overweight/obese patients relative to normal-weight patients.

Recently, Vora *et al*. utilized cephalometric and geometric morphometric analyses to study facial forms among obese and normal-weight adolescents in a matched sample (*n* = 24 per group) [18]. Initially, they found greater maxillary length, mandibular prognathism, and a brachycephalic facial form among pre-treatment records of growing obese patients [18]. However, upon application of Bonferroni correction, both maxillary and mandibular lengths fail to have a significant difference. In our sample, we found elevated maxillary and mandibular lengths among overweight adolescents, although we did not observe mandibular prognathism nor a brachycephalic facial form, in our initial and final records of overweight/obese patients. In a study evaluating pre-treatment cephalometric radiographs, Gordon *et al.* also did not observe mandibular prognathism among overweight/obese patients, similar to our findings [19]. These differences from Vora *et al.* may stem from varying methodologies and markedly disparate sample sizes (our sample: *N* = 326 total and Gordon *et al*: *N* = 181 total versus Vora *et al*: *N* = 48 total) [18, 19].

Several significant linear correlations were found between craniofacial measures and BMI percentile. Notably, mandibular unit length (Ar-Gn) had a change of 0.66 mm, maxillary unit length (Co-ANS) had a change of 0.33 mm, and lower face height (ANS-Me) had a change of 0.38 mm, for every 10% change in BMI percentile (Table 4). As BMI increased, there was also a linear increase in the LFH:total FH percentage with a concomitant decrease in UFH:total FH percentage, consistent with findings in Ohrn *et al.* [17]. This paired increase in LFH:total FH percentage with a concomitant decrease in UFH:total FH percentage is seen in the overweight/obese cohort as they present with greater maxillary and mandibular sizes, leading to a greater vertical dimension in the lower face. There was negligible change in measures of mandibular plane angle including SN-GoGn and SN-Pg before and after treatment in both cohorts, indicating that the observed increase in LFH:total FH percentage was growth related and not due to vertical treatment effects (i.e. clockwise mandibular autorotation) (SN-GoGn: decreased 0.1° on average (*P* = 0.417), normal weight −0.2°, overweight +0.3° (*P* = 0.111); SN-Pg: decreased 0.3° on average (*P* = 0.067), normal weight +0.5°, overweight +0.1° (*P* = 0.102)) (Supplementary Tables S2 and S3) [10].

In our sample in Danze *et al.* and in studies by Mack *et al.*, Hilgers *et al.*, and Olszewska *et al.* researchers observed earlier initiation of the pubertal growth spurt in overweight/obese patients with more advanced CVM and dental developmental staging [8–11]. Though the growth spurt begins earlier, post-treatment data evaluated here indicate that growth does not progress at a faster rate during treatment in adolescents with elevated BMI compared to normal weight patients, nor does the earlier initiation of growth result in earlier cessation of growth in this group. There was no significant difference in the average treatment length, the average age at treatment completion and the CVM stage distribution at treatment conclusion between the normal and overweight/obese BMI groups (Table 2). Steady growth appears to occur over a longer period of time in obese/overweight patients, resulting in the larger post-treatment dimensions observed in this cohort (Fig. 1, Table 3). As one of the only studies to evaluate pre- and post-treatment cephalometric outcomes, we are the first to find no significant difference in the rate of growth during treatment between normal and overweight/obese adolescents.

Mounting evidence suggests that elevated BMI impacts multiple aspects of development in children including effects on dentition, bone growth and maturation. Recent studies demonstrate that obesity is associated with advanced dental age, earlier emergence of teeth, and a dental development rate that increases with age suggesting that elevated BMI accelerates dental maturation [8–10, 20, 21]. Our study of pre-treatment records also identified more advanced dental ages among overweight/obese children, but *during treatment*, we found no difference in the rate of craniofacial growth between BMI cohorts, unlike dental development as described by Nicholas *et al.* [10, 20]. Dental development studies use the Demirjian staging method (based on scoring dental calcification), while facial growth rates are determined by measuring exact skeletal dimensions on cephalograms; this difference in methodology confounds our ability to draw direct comparisons between craniofacial and dental development rates [22]. Additionally, Nicholas *et al.* evaluated dental development over a younger range of discrete ages (4, 8, and 12 years old) which differs from our older, continuous cohort (T1: 8–14 years old; T2: 12–18 years old) [20].

Cephalometric studies have found advanced cervical skeletal maturation associated with elevated BMI in pre-treatment records [9, 10]. Similarly, in this study, the elevated BMI group had significantly advanced CVM staging compared to the normal weight cohort pre-treatment, but by treatment end, there was no difference in CVM between groups (Table 2). When looking at the appendicular skeleton, obese children tend to be taller than normal-weight peers, but this difference in height does not persist into adulthood [23–26], suggesting that the increase in childhood statural growth is due to earlier skeletal maturation rather than total height gains in overweight kids. However, data presented here support an earlier initiation and longer period of craniofacial growth, resulting in larger final facial dimensions among overweight/obese children. The biological causes underlying these differences in craniofacial and statural growth are unknown, but could be a valuable area of future inquiry. Altogether our findings are consistent with the literature indicating that hard tissue growth and maturation are impacted by elevated BMI, with some features unique to the developing dentition, craniofacial, and appendicular skeletons. Future investigation could be aimed at understanding the biological mechanisms underlying accelerated dental, cranial, and statural development and increased facial dimensions seen in overweight adolescents.

Study limitations include differences in racial and ethnic distribution between cohorts, with a higher representation of African Americans and Hispanics in the overweight/obese cohort; this could influence the overweight/obese groups’ craniofacial characteristics and values. As a result, statistical adjustments were made for race and ethnicity. Patients with a CVM of 5 were included in the study and may still have slight growth remaining, compared to participants with a CVM of 6. The age ranges of both cohorts were sizable, and larger standard deviations were observed for some craniofacial dimensions, likely related to this age heterogeneity within the cohorts; however, between the two cohorts, there was no significant difference in mean age and range at treatment start T1 and finish T2. An additional limitation is that BMI was collected only with pre-treatment records, and not at mid-treatment nor during final records. As a result, we could not account for any change in BMI during treatment, between initial and final records. Although mid- and post-treatment BMI was unknown, an increased BMI at the start of pubertal growth is likely to persist throughout orthodontic treatment [27]. Sandeep *et al.* investigated changes in BMI during the first 3 months of orthodontic treatment and found that after a brief decrease in BMI during month 1, BMI rebounded and was not significantly different thereafter [27]. Finally, we have established that the same amount of growth occurred in both cohorts over the course of treatment, such that the average growth rate is equivalent, but we cannot exclude the possibility that the growth rate differed between groups at particular time points during treatment.

Our findings underline the impact of BMI on craniofacial development, indicating that providers should collect weight and height data, to factor BMI into their treatment planning and timing. Results suggest that orthodontic treatment could begin earlier in overweight/obese patients to correspond with their initiation of growth. The period of growth for patients with elevated BMI appears to be longer, providing a lengthier window of treatment time for growth modification. Orthodontic providers can also anticipate a more bimaxillary prognathic presentation among patients with elevated BMI. With the high prevalence of adolescent obesity, it is important for orthodontic and paediatric providers to be aware of the impact of elevated BMI on craniofacial development and timing for optimal clinical care.

## Conclusions

- Craniofacial growth begins earlier in overweight/obese children than normal-weight patients and continues throughout treatment at a rate similar to normal-weight adolescents. Adolescents with ­normal weight and elevated BMI grew during treatment, with no difference in the amount of cephalometric change. Regression analyses show no linear correlation between change in growth during treatment and BMI.
- The longer period of growth in patients with elevated BMI results in larger skeletal dimensions at the treatment end. Craniofacial dimensions of overweight/obese adolescents are consistent with a proportional, bimaxillary prognathic presentation, with clinically significant increases in maxillary and mandibular length compared to normal-weight patients.
- BMI percentile was a significant linear predictor for cephalometric post-treatment outcomes, including Ar-Gn, Co-ANS, ANS-Me, UFH:total FH (inverse relationship), LFH:total FH, SNA, and SNB. Orthodontic providers can anticipate a more bimaxillary prognathic presentation among patients with elevated BMI.
- Orthodontic treatment could begin earlier in overweight/obese patients to correspond with the beginning of their growth. The period of growth for patients with elevated BMI appears to be longer, providing an increased window for growth modification.

## Supplementary Material

cjad043_suppl_Supplementary_Tables

## Data Availability

The data that support the findings are included in the Supplementary materials. Any further information is available from the corresponding author upon reasonable request.
