# Supplementary material for: Influence of BMI percentile on craniofacial morphology and development in adolescents,Part II: elevated BMI is associated with larger final facial dimensions
Source: Eur J Orthod. 2023 Nov 2;46(1):cjad043. doi: 10.1093/ejo/cjad043 (PMC10783153; doi:10.1093/ejo/cjad043)
Supplement: cjad043_suppl_Supplementary_Tables [file cjad043_suppl_supplementary_tables.docx]

**Supplemental Tables**

**Supplemental Table 1.** Intra-examiner reliability based on Rho C concordance correlation coefficients

| Variable* | rho C^ | 95% CI |
| --- | --- | --- |
| Ar-Gn (mm) | 1.00 | 1.00-1.00 |
| Go-Pg (mm) | 0.98 | 0.96-0.99 |
| Co-ANS (mm) | 0.98 | 0.96-0.99 |
| PNS-ANS (mm) | 0.94 | 0.89-0.97 |
| S-N (mm) | 0.97 | 0.95-0.99 |
| S-Go (mm) | 1.00 | 0.99-1.00 |
| N-Me (mm) | 0.99 | 0.98-0.99 |
| N-ANS (mm) | 0.94 | 0.88-0.97 |
| ANS-Me (mm) | 1.00 | 0.99-1.00 |
| UFH:totalFH | 0.97 | 0.95-0.99 |
| LFH:totalFH | 0.96 | 0.93-0.98 |
| SNA (deg) | 0.93 | 0.87-0.96 |
| SNB (deg) | 0.95 | 0.91-0.97 |
| ANB (deg) | 0.98 | 0.97-0.99 |
| SN-Pg (deg) | 0.96 | 0.92-0.98 |
| SN-GoGn (deg) | 0.99 | 0.97-0.99 |

*Variable abbreviations: Ar, articulare; Gn, gnathion; Go, gonion; Pg, pogonion; Co, condylion; ANS, anterior nasal spine; PNS, posterior nasal spine; S, sella; N, nasion; Me, menton; UFH, upper face height; LFH, lower face height; SNA, sella-nasion-A point; SNB, sella-nasion-B point; ANB, A point-nasion-B point.

^Rho C Concordance values are measured from 0.00-1.00. The closer to 1.00, the better the reliability.  A Rho C value of 0.90 or greater is considered excellent reliability.

**Supplemental Table 2.** Mean change in craniofacial dimensions between T1 and T2 by weight group

|  | Normal Weight  BMI Group  (n=158) | | | | Overweight/Obese  BMI Group  (n=168) | | | |  | | |
| --- | --- | --- | --- | --- | --- | --- | --- | --- | --- | --- | --- |
| Variable | Mean  Change | SD | Min | Max | Mean  Change | SD | Min | Max | p-value | Bonf. P-value | Mean Diff.** |
| Ar-Gn (mm) | 4.1 | 5.5 | -13.8 | 24.2 | 5.0 | 11.9 | -11.3 | 104.7 | 0.579 | 1.000 | -0.57 |
| Go-Pg (mm) | 2.4 | 3.0 | -3.3 | 13.5 | 3.0 | 3.1 | -2.1 | 11.9 | 0.082 | 1.000 | -0.59 |
| Co-ANS (mm) | 1.8 | 4.5 | -17.1 | 23.7 | 1.7 | 4.5 | -36.1 | 13.3 | 0.678 | 1.000 | 0.21 |
| PNS-ANS (mm) | 3.8 | 3.6 | -7.0 | 16.1 | 4.1 | 3.0 | -5.4 | 14.3 | 0.386 | 1.000 | -0.32 |
| S-N (mm) | 1.5 | 2.3 | -6.2 | 15.3 | 1.4 | 1.9 | -5.3 | 6.6 | 0.564 | 1.000 | 0.13 |
| S-Go (mm) | 4.9 | 5.0 | -15.6 | 22.2 | 3.8 | 4.2 | -9.4 | 15.1 | 0.029* | 0.464 | 1.02 |
| N-Me (mm) | 5.5 | 4.8 | -13.5 | 28.8 | 5.2 | 4.7 | -10.7 | 15.8 | 0.643 | 1.000 | 0.22 |
| N-ANS (mm) | 2.0 | 2.8 | -8.3 | 13.1 | 1.8 | 2.8 | -10.2 | 12.3 | 0.282 | 1.000 | 0.32 |
| ANS-Me (mm) | 3.3 | 3.1 | -4.9 | 17.1 | 3.2 | 2.8 | -6.0 | 10.7 | 0.988 | 1.000 | 0.00 |
| UFH:total FH | -0.004 | 0.014 | -0.052 | 0.043 | -0.005 | 0.016 | -0.100 | 0.057 | 0.375 | 1.000 | 0.001 |
| LFH:total FH | 0.002 | 0.016 | -0.052 | 0.047 | 0.002 | 0.015 | -0.062 | 0.057 | 0.725 | 1.000 | -0.001 |
| SNA (deg) | -0.3 | 2.2 | -10.6 | 7.5 | -0.6 | 2.2 | -8.2 | 8.7 | 0.265 | 1.000 | 0.28 |
| SNB (deg) | 0.2 | 2.0 | -7.6 | 7.6 | -0.1 | 2.0 | -10.0 | 5.1 | 0.209 | 1.000 | 0.28 |
| ANB (deg) | -0.5 | 1.7 | -6.0 | 8.7 | -0.4 | 1.5 | -5.1 | 4.1 | 0.970 | 1.000 | -0.01 |
| SN-Pg (deg) | 0.5 | 2.0 | -7.2 | 7.0 | 0.1 | 2.0 | -8.9 | 5.5 | 0.102 | 1.000 | 0.37 |
| SN-GoGn (deg) | -0.2 | 2.5 | -7.1 | 8.4 | 0.3 | 2.5 | -5.4 | 10.6 | 0.111 | 1.000 | -0.45 |

*Indicates significance (p-value<0.05) by ANOVA prior to multiple testing adjustment.  Please interpret at your discretion.

**A negative mean difference indicates that the overweight/obese individuals had a larger change in mean value than the normal weight individuals.

**Supplemental Table 3.** Mean change in craniofacial dimensions between T1 and T2 for the entire cohort (all patients, all weights)

| Variable | Mean  Change | SD | Min | Max | P-value | Bonf. P-value |
| --- | --- | --- | --- | --- | --- | --- |
| Ar-Gn (mm) | 4.6 | 9.4 | -13.8 | 104.7 | <.001* | <.002** |
| Go-Pg (mm) | 2.7 | 3.1 | -3.3 | 13.5 | <.001* | <.002** |
| Co-ANS (mm) | 1.7 | 4.5 | -36.1 | 23.7 | <.001* | <.002** |
| PNS-ANS (mm) | 4.0 | 3.3 | -7.0 | 16.1 | <.001* | <.002** |
| S-N (mm) | 1.5 | 2.1 | -6.2 | 15.3 | <.001* | <.002** |
| S-Go (mm) | 4.3 | 4.6 | -15.6 | 22.2 | <.001* | <.002** |
| N-Me (mm) | 5.4 | 4.7 | -13.5 | 28.8 | <.001* | <.002** |
| N-ANS (mm) | 1.9 | 2.8 | -10.2 | 13.1 | <.001* | <.002** |
| ANS-Me (mm) | 3.3 | 2.9 | -6.0 | 17.1 | <.001* | <.002** |
| UFH:total FH | -0.0044 | 0.015 | -0.010 | 0.057 | 0.170 | 1.000 |
| LFH:total FH | 0.0020 | 0.015 | -0.062 | 0.057 | 0.561 | 1.000 |
| SNA (deg) | -0.4 | 2.2 | -10.6 | 8.7 | 0.197 | 1.000 |
| SNB (deg) | 0.0 | 2.0 | -10.0 | 7.6 | 0.048 | 0.768 |
| ANB (deg) | -0.4 | 1.6 | -6.0 | 8.7 | 0.467 | 1.000 |
| SN-Pg (deg) | 0.3 | 2.0 | -8.9 | 7.0 | 0.067 | 1.000 |
| SN-GoGn (deg) | 0.1 | 2.5 | -7.1 | 10.6 | 0.417 | 1.000 |

*Indicates significance (p-value<0.05) by ANOVA prior to multiple testing adjustment.  Please interpret at your discretion.

**Indicates significance (p-value<0.05) after Bonferroni adjustment.

**Supplemental Table 4.** Significant linear regression slopes for the change in craniofacial dimensions as a function of BMI percentile

| Variable | Slope | P-value | Bonf. P-value |
| --- | --- | --- | --- |
| Ar-Gn (mm) | 0.07 | 0.722 | 1.000 |
| Go-Pg (mm) | 0.10 | 0.128 | 1.000 |
| Co-ANS (mm) | -0.08 | 0.381 | 1.000 |
| PNS-ANS (mm) | 0.04 | 0.568 | 1.000 |
| S-N (mm) | -0.07 | 0.088 | 1.000 |
| S-Go (mm) | -0.20 | 0.026* | 0.416 |
| N-Me (mm) | -0.12 | 0.210 | 1.000 |
| N-ANS (mm) | -0.12 | 0.048* | 0.768 |
| ANS-Me (mm) | -0.03 | 0.599 | 1.000 |
| UFH:total FH | -0.0005 | 0.146 | 1.000 |
| LFH:total FH | 0.0002 | 0.498 | 1.000 |
| SNA (deg) | -0.01 | 0.791 | 1.000 |
| SNB (deg) | -0.01 | 0.810 | 1.000 |
| ANB (deg) | -0.00 | 0.933 | 1.000 |
| SN-Pg | -0.03 | 0.464 | 1.000 |
| SN-GoGn (deg) | 0.04 | 0.474 | 1.000 |

*Indicates significant linear relationship between craniofacial dimension and BMI% (p-value<0.05) prior to multiple testing adjustment.  Please interpret at your discretion.
